# Exploring association between certified EHRs adoption and patient experience in U.S. psychiatric hospitals

**DOI:** 10.1371/journal.pone.0234607

**Published:** 2020-06-17

**Authors:** Xuejun Hu, Haiyan Qu, Shannon H. Houser, Jingmei Ding, Huoliang Chen, Xianzhi Zhang, Min Yu

**Affiliations:** 1 Department of Health Services Administration, Air Force Medical University, Xi’an, China; 2 Department of Health Services Administration, University of Alabama at Birmingham, Birmingham, AL, United States of America; 3 Department of Health Services Administration, Academy of Military Medical Sciences, Beijing, China; University of Toronto, CANADA

## Abstract

**Objective:**

Certified Electronic Health Records (EHR) have been shown to improve the health service quality in some health settings, but there is scant evidence related to its adoption in psychiatric hospitals. This paper aimed to examine the relationship between certified EHR adoption and patient experience across psychiatric hospitals in the United States.

**Methods:**

A cross-sectional study design compared the difference in patient experience measures between psychiatric hospitals with and without certified EHR. Data were drawn from the American Hospital Association (AHA) Annual Survey Database and Hospital Compare datasets. Eleven publicly reported measures for patient experience from the Consumer Assessment of Healthcare Providers and Systems Hospital Survey (HCAHPS) were applied for analysis. Independent relationship of certified EHR adoption and patient experience was explored with multiple linear regression models adjusted for hospital organizational characteristics.

**Results:**

Positive associations were identified between certified EHR adoption and five patient perception measures—“recommend hospital” (β = 0.66, 95% CI = [0.16,1.16]; t = 2.68, p = 0.010), “overall hospital rating” (β = 0.39, 95% CI = [0.03,0.75]; t = 2.11, p = 0.035), “discharge information” (β = 0.45, 95% CI = [0.03,0.86]; t = 2.09, p = 0.037), “care transition” (β = 0.44, 95% CI = [0.14, 0.75]; t = 2.84, p = 0.005), and “responsiveness of hospital staff” (β = 0.47, 95% CI = [0.04, 0.90]; t = 2.13, p = 0.033).

**Conclusion:**

Our results suggest the positive association between certified EHR adoption and patient experience. More studies are needed to explore impacts of certified EHR adoption and potential improvement in patient experience to quality of care.

## Introduction

Enacted by Health Information Technology for Economic and Clinical Health (HITECH) Act in 2009, Electronic Health Records (EHR) Incentive Programs have been launched to drive nationwide EHR adoption and meaningful use of health information technology throughout health care settings in the United States (U.S.). [1] An unprecedented progress has been made in utilization of certified EHR in U.S. health settings and large numbers of studies have demonstrated its substantial influences on the quality, safety, and efficiency of health services. [2–4] However, psychiatric hospitals are ineligible for the financial incentive programs, and the rate of EHR adoption is noticeably lower among psychiatric hospitals [5, 6] compared to other hospitals. While more than 80 percent of general hospitals adopted EHR by 2015, only 15 percent of psychiatric hospitals adopted at least a basic EHR system produced by different vendors. [7] There might be multiple barriers impeding adoption of certified EHR, including initial financial pressure of providers, narrative and non-structure feature of patient records, confidentiality of psychiatric care and stigma of mental illness. [8, 9] Lag in certified EHR adoption among psychiatric hospitals might not only limit quality improvement of psychiatric care but also stymie efforts to achieve the targeted benefits, such as interoperability, across the health care continuum. [6, 10] Therefore, it is necessary to identify the evidence to prove if certified EHR adoption has positive impacts to quality improvement and patient experience in psychiatric hospitals, and also if health policymakers should expand the incentive programs to psychiatric hospitals.

According to prior published studies conducted in psychiatric settings, positive impacts of certified EHR utilization can be found on the therapeutic communications, [11, 12] hospital readmission, [13] adverse drug events [14] and psychiatrist-patient relationship [15–17] as well as other quality measures [18–20] based on psychiatric patient or practitioner level instead of hospital level. A gap was identified with very limited literature in the relationship between patient experience and certified EHR adoption in psychiatric care using nationally representative data.

As more emphasis is placed on the concept of patient-centered care, patient experience has become the integral component to evaluate the health quality. Some studies have examined the impact of the EHR adoption on the patient satisfaction in general health settings. [21, 22] However, previous findings about such relationship are mixed. Some studies suggested that EHR use could significantly improve patient experience [23–25] whereas other showed insignificant [26–30] or even inverse associations. [31, 32] Additionally, most literature on the relationship between patient experience and EHR adoption were conducted in non-psychiatric settings. [9, 22–28, 30] Due to the confidentiality and sensitive nature in psychiatric records, and the special reliance on information for psychiatric diagnosis and treatment, the findings of previous works in non-psychiatric hospitals may not apply equally to psychiatric hospitals. [17]

The aim of this cross-sectional study is to examine the relationship between certified EHR adoption and patient experience at hospital level in psychiatric care setting in the U.S. We hypothesized that certified EHR use would positively influence the patient experience across psychiatric hospitals.

## Methods

### Data source and sample

This study was a cross-sectional, secondary analysis based on data from two primary databases which were open and publicly available: American Hospital Association (AHA) Annual Survey Database and the Centers for Medicare and Medicaid Services (CMS) Hospital Compare datasets. AHA Annual Survey Database provides the characteristics information for 6,251 hospitals, including teaching status, bed size, metropolitan status, ownership, system membership status, primary service classification, and length of stay. Hospital Compare datasets include information on the patient experience from the Consumer Assessment of Healthcare Providers and Systems Hospital Survey (HCAHPS) conducted in 4,806 hospitals during the period from April 1^st^ 2016 to March 31^st^ 2017. The HCAHPS survey is administered to a random sample of adult patients across medical conditions between 48 hours and 6 weeks after discharge. Hospital Compare datasets provide data on the adoption of certified EHR from the Inpatient Psychiatric Facility Quality Reporting (IPFQR) Program in 2016, with data for 1,655 psychiatric hospitals.

Using the Medicare identification number issued by the Medicare Administrative Contractor's (MAC) Provider Enrollment Department to a unique provider, data from different sources were merged into a master dataset. For the purpose of better assess the reliable of hospital performance from the HCAHPS survey, 649 hospitals that received fewer than 100 responses to HCAHPS surveys were excluded from the total 1,655 psychiatric hospitals in our analysis. The final study sample included 1,006 psychiatric hospitals with total 1,101,140 unique patients involved as respondents to the HCAHPS surveys from those hospitals. Because the information was anonymous and no personal information was collected, this study was exempt from requirement for institutional review board approval.

### Certified EHR adoption and cohorts

In IPFQR Program, there was a structural measure evaluating the degree to which hospitals adopted certified EHR in health services. Hospitals were required to attest to one of three statements that best represented hospital’s highest typical adoption of EHR: 1) Certified EHR technology (certified under the Office of the National Coordinator(ONC) for Health Information Technology (HIT) Certification Program) is employed most commonly to exchange health information at times of transitions in care; 2) Non-Certified EHR Technology (that is, not certified under the ONC HIT Certification Program) is used most commonly to transfer health information at times of transitions in care; 3) Paper or Other Form (for example, email) is the most common approach to conduct information exchange not involving the transfer of health information using EHR technology at times of transitions in care. The psychiatric hospitals choosing the first statement were included into observation group defined as hospitals with Certified EHR, whereas those responding with the second or third statement were categorized into a control group defined as hospitals with non-certified EHR. Only 20 hospitals using “non-certified EHR” in our dataset, which we combined with those hospitals using “Paper or Other Form” into the control group as “Paper-based/Non-Certified” (refers to without certified EHR).

### Outcome measures of patient experience

There were 11 publicly reported measures for patient experience in HCAHPS database: communication with nurses, communication with doctors, responsiveness of hospital staff, pain management, communication about medicines, discharge information, care transition, cleanliness of hospital environment, quietness of hospital environment, overall hospital rating and recommend the hospital. Each measure was constructed from an individual item or composited based on two/three items on the HCAHPS survey which includes 25 items in total. A 0–100 linear-scaled score (“Linear Score”) for each measure was calculated using a set of algorithms based on the responses to the survey items. [33, 34] Details regarding the specifics of how these measures were constructed and how the “Linear Score” were calculated are available online at www.hcahpsonline.org/en/hcahps-star-ratings/.

We hypothesize that there will be positive associations between certified EHR adoption and those measures related to information communication: communication with nurses, communication with doctors, responsiveness of hospital staff, communication about medicines, discharge information, care transition, overall hospital rating and recommend the hospital.

### Hospital characteristics

All included hospitals were community hospitals, long-term hospitals, and acute care hospitals that provided inpatient medical care. Hospitals affiliated to a certain health care system were coded as “system affiliation” (“1”) and other hospitals coded as “non-system affiliation” (“0”). Teaching status included non-teaching (“0”) and teaching (“1”) that combined major and micro teaching hospitals defined in AHA. Hospital locations were divided into rural area (“0”) and urban area (“1”). Bed-size was coded as small (< 200 beds, coded as “0”), medium (200–400 beds, “1”), and large (> 400 beds, “2”). Hospital ownership was classified as government (coded as “0”), non-profit (“1”), and for-profit (“2”).

### Data analysis

Independent samples t-tests were employed to examine the statistic differences in patient experience between certified EHR adoption (coded as “1”) and paper-based/non-certified EHR (coded as “0”). However, previous research have indicated that patient experience may potentially be correlated to hospital characteristics including ownership, [35, 36] hospital location, [36, 37] hospital bed size, [38, 39] and teaching status [40]. To determine independent association between certified EHR adoption and outcome of patient perception, variables about hospital characteristics were entered into a multiple linear regression model that also accounted for significant correlations between variables. The regression model is given below/ was as follows.

Patient experience = ƒ(certified EHR adoption, bed size, urban location, teaching status, ownership, affiliation).

All p values were 2-tailed and p < 0.05 was considered statistically significant. Data analyses were conducted using IBM SPSS statistical software program, version 24.0 (IBM SPSS, 2017).

## Results

### Hospital characteristics and certified EHR adoption

Hospital organizational characteristics, presented by certified EHR adoption status, are summarized in Table 1. Of the 1,006 sampled psychiatric hospitals in the U.S., the majority were affiliated with the healthcare systems (71.3%), located in urban area (77.8%), and belong to teaching hospitals (61.7%); slightly more than half (51.4%) were non-government & non-profit entities. The majority (74.1%) had less than 400 beds.

**Table 1:** Hospital characteristics by EHR status.

		All (n = 1,006)	Certified EHR (n = 564)	Paper-based/Non-Certified EHR (n = 442)	p-value
**Dependent variables,** Mean(SD) ^##^					
Responsiveness of hospital staff		84.0(3.8)	84.0(3.6)	83.9(4.0)	0.561
Discharge information		86.7(3.4)	86.9(3.2)	86.5(3.6)	0.084
Care transition		80.7(2.5)	81.0(2.4)	80.4(2.6)	0.002
Overall hospital rating		88.1(2.9)	88.3(2.8)	87.8(3.0)	0.013
Recommend hospital		87.2(4.1)	87.7(4.1)	86.6(4.2)	<0.001
**Independent variables,** N(%)^#^					
Ownership*	Government	157(21.5)	88(15.6)	69(15.7)	0.043
	Non-profit	637(51.4)	374(66.3)	263(59.9)	
	For profit	209(27.1)	102(18.1)	107(24.4)	
Teaching status	No	385(38.3)	190(33.7)	195(44.1)	0.001
	Yes	621(61.7)	374(66.3)	247(55.9)	
System affiliation	No	289(28.7)	144(25.5)	145(32.8)	0.011
	Yes	717(71.3)	420(74.5)	297(67.2)	
Location*	Rural	223(22.2)	106(18.8)	117(26.7)	0.003
	Urban	780(77.8)	458(81.2)	322(73.3)	
Bed size*	Small	415(41.4)	203(36.0)	212(48.3)	<0.001
	Medium	328(32.7)	184(32.6)	144(32.8)	
	Large	260(25.9)	177(31.4)	83(18.9)	

Abbreviations: EHR, electronic health records.

^#^ Pearson χ^2^ tests for categorical variables.

^##^ Independent-samples t tests were conducted to compare the difference in means of measures for patient perception of interoperability.

* Missing values

Notes: Results related to 6 variables, including communication with nurses, communication with doctors, pain management, communication about medicines, cleanliness of hospital environment and quietness of hospital environment, were not presented in this paper because no significant associations had been found between them and certified EHR adoption.

Among the 1,006 hospitals, 564 (56.1%) have adopted certified EHR technology. There was significant difference in ownership type (p = 0.043), teaching status (p = 0.001), system affiliation (p = 0.011), bed-size (p <0.001), and location (p = 0.003) between hospitals with and without certified EHR adoption.

### Association of patient experience and certified EHR adoption

Among the 11 measures of patient experience, only two measures (“cleanliness” and “doctor communication”) saw subtly but non-significantly lower scores in hospitals with certified EHR, whereas all other measures scored slightly higher in univariate analyses. Hospitals with certified EHR had significantly higher average scores in three measures of “recommend hospital” (Mean difference = 1.05; t = 4.0, p<0.001), “overall hospital rating’ (Mean difference = 0.46; t = 2.5, p = 0.013), and “care transition” (Mean difference = 0.38; t = 3.1, p = 0.002) (Table 1). In addition, Pearson’s correlation analysis showed associations between certified EHR adoption and measures of patient experience including “care transition”, “discharge information”, “responsiveness of hospital staff”, “overall hospital rating” and “recommendation” (Table 2).

**Table 2:** Correlations between Hospital characteristics and EHR status.

	Care transition	Discharge information	Responsiveness of hospital staff	Overall hospital rating	Recommendation	1	2	3	4	5	6	7
1 System affiliation	-0.087*	-0.049*	-0.093*	-0.029*	-0.022*							
2 Non-profit	0.248**	0.169**	0.160**	0.139**	0.219**	-0.025						
3 For-profit	-0.319**	-0.149**	-0.161**	-0.175**	-0.272**	0.286**	-0.677**					
4 Urban	-0.102**	-0.155**	-0.327**	-0.069*	0.088*	0.204**	0.063*	0.026				
5 Teaching status	-0.016*	-0.096**	-0.250**	-0.029*	0.127**	0.073*	0.177**	-0.164**	0.405**			
6 200–400 beds	-0.031*	-0.042*	-0.093*	-0.024*	0.019*	0.035	0.052	0.014	0.184**	0.153**		
7 >400 beds	0.019*	-0.074*	-0.219**	0.039*	0.169**	0.066*	0.066*	-0.147**	0.300**	0.403**	-0.412**	
8 Certified EHR	0.100**	0.054*	0.021*	0.078*	0.123**	0.075*	0.066*	-0.077*	0.094**	0.103**	-0.002	0.141**

*P value<0.05

**P value<0.001

Table 3 presents the results from multiple linear regression that test the association between patient experience and certified EHR adoption. Controlled for hospital organizational characteristics, results from the linear regression analysis indicated that there was a significant association between certified EHR adoption and five satisfaction measures: “discharge information” (β = 0.45, 95% CI = [0.03,0.86]; t = 2.09, p = 0.037), “care transition” (β = 0.44, 95% CI = [0.14, 0.75]; t = 2.84, p = 0.005), “responsiveness of hospital staff” (β = 0.47, 95% CI = [0.04, 0.90]; t = 2.13, p = 0.033), “recommend hospital” (β = 0.66, 95% CI = [0.16,1.16]; t = 2.68, p = 0.010), and “overall hospital rating” (β = 0.39, 95% CI = [0.03,0.75]; t = 2.11, p = 0.035). These results almost remained consistent with those from univariate analysis.

**Table 3:** Regression results of patient experience measures.

Independent variable	Care transition, Coef.(95% CIs)	Discharge information, Coef.(95% CIs)	Responsiveness of hospital staff, Coef.(95% CIs)	Recommendation, Coef.(95% CIs)	Overall hospital rating, Coef.(95% CIs)
Certified EHR ^a^	0.44*(0.14~0.75)	0.46*(0.03~0.86)	0.47*(0.03~0.86)	0.66*(0.16~1.16)	0.39*(0.03~0.75)
System affiliation ^b^	0.04(-0.33~0.40)	0.00(-0.50~-0.50)	0.04(-0.50~0.50)	0.16(-0.43~0.75)	0.15(-0.28~0.58)
Location-urban ^c^	-0.56*(-0.98~-0.14)	-0.97**(-1.55~-0.40)	-1.94**(-1.55~-0.40)	0.24(-0.45~0.93)	-0.53*(-1.03~-0.03)
Teaching status ^d^	-0.25(-0.63~0.12)	-0.42(-0.93~0.10)	-0.90**(-0.93~0.10)	-0.07(-0.69~0.54)	-0.42(-0.87~0.03)
Bedsize-middle ^e^	-0.03(-0.42~0.37)	-0.31(-0.84~0.23)	-0.97**(-0.84~0.23)	0.68*(0.04~1.32)	0.17(-0.30~0.63)
Bedsize-large ^f^	0.04(-0.42~0.50)	-0.46(-1.09~0.17)	-1.65**(-1.09~0.17)	1.41**(0.66~2.17)	0.44(-0.11~0.99)
Ownership-non-profit ^g^	0.40(-0.38~0.83)	1.06**(0.46~1.66)	1.10**(0.46~1.66)	0.47(-0.24~1.19)	0.30(-0.22~0.82)
Ownership-profit ^h^	-1.68**(-2.23~-1.14)	-0.48(-1.23~0.27)	-0.95*(-1.23~0.27)	-2.19**(-3.1~-1.29)	-1.03*(-1.68~-0.38)
R^2^	0.12	0.07	0.19	0.11	0.05

*P value<0.05

**P value<0.001

a: reference group is Paper-based/Non-Certified EHR

b: reference group is Non- system affiliation

c: reference group is Location-rural

d: reference group is Non- teaching status

e, f: reference group is Bedsize-small

g, h: reference group is Ownership-government.

The adjusted R^2^ values in all the five regression models indicated that those regression models had explained 4% to 22% of variance in each patient experience measure respectively (Table 3). In multivariate analysis, all multiple linear regression models had significant F tests, indicating the overall significance of the models.

## Discussion

There is widespread agreement that the certified EHR is a lever to the care quality among hospitals which theoretically is helpful to improve service process and result in better patient experience. [9, 41] Empirical evidence is needed to testify about the implications of certified EHR use on the patient experience in psychiatric hospitals. This study sought to explore association between certified EHR adoption and patient experience in U.S. psychiatric hospitals by assessing the disparities in patient perception between psychiatric hospitals with and without certified EHR. Results in this study revealed that certified EHR adoption is positively and significantly associated with several categories of patient experience elements.

Our results reveal that a higher level of patient satisfaction with care transition was found in psychiatric hospitals which have adopted of certified EHRs. According to the HCAHPS survey, highly scored satisfaction with care transition means that patients could understand better about their care when leaving hospitals, such as clearly understanding the purpose for taking each of their medications and their own responsibilities for managing their health. The implementation of certified EHR could lead to improvements in care information availability for patients and/or their families, [19, 42] which may be beneficial and helpful for patients to better manage their care. Certified EHR may also help mental health providers take patients’ preferences (and those of their families or caregivers) into account in deciding what patients’ health care needs would be when patients were discharged [25]. In addition, certified EHR advance the data interoperability and promote sharing of information among providers, which potentially improve service coordination across different settings. [8]

We also identified positive relationships between patient satisfaction with discharge information in hospitals adopting certified EHR. Compared to the information provided in a paper format, certified EHR adoption proved to be a more accurate, accessible and safer form of communication between patients and providers. It is critical for psychiatric patients and their families/caregivers to receive information about their health conditions and patient’s care plan at the discharge. Safety features of certified EHR, such as storability of data and spell check, may provide patients with easier access to the correct health information after discharge. [25] In contrast, patients may be likely to be worried about illegibility of handwriting and reading information correctly in paper health records. [25, 43, 44] Also, EHR systems can provide easy and quick access to patients’ data, such as test results and billings for multiple services and strategies, which is useful to improve availability of discharge information for patients.

Our study results showed that certified EHR adoption is positively related to the patient perception of the responsiveness of hospital staff. On the one hand, staff may be free from the manual task with digital technology and have more time for the attention to patient progress and subsequent problem solving; [45] on the other hand, certified EHR may assist mental health practitioners timely tracking the patient situation and behavior changes, manage risk with incident notification, and automatically remind staff of the upcoming events in the care plan [9]. All the efficient interaction between patients and mental health practitioners could potentially reduce their waiting time for services and thus improve their patient experience with responsiveness.

Furthermore, hospitals with certified EHR embraced higher overall patient experience and were more likely to be recommended by patients to their friends and family members. These two comprehensive measures, “recommend hospital” and “overall hospital rating”, were more robust to totally demonstrate the positive effect of certified EHR on the patient satisfaction than those individual measures above which showed consistent positive results.

Despite of results mentioned above, patient experience with interpersonal care, including doctor and nurse communication were not found significant difference between with and without certified EHR. These results were consistent with previous works that there was no relationship between EHR adoption and patient perceptions about patient-doctor communication. [30, 46, 47] Some prior relevant work even found that patient-doctor communication may be negatively impacted by EHR adoption, possibly due to less experience and comfort of mental health practitioners incorporating EHR technology into patient care, [25, 31, 48] as well as distraction to patients and reduction in eye-contact. [32] Those neutral or negative results remind that interpersonal care should be a focus to reduce the unexpected adverse effects of certified EHR adoption in psychiatric hospitals [49] as communication skills and psychodynamic interpretations are arguably more highlighted in psychiatric hospitals than in non-psychiatric settings.

In all, positive findings in this paper should lessen the concerns of psychiatric hospitals over potentially patient dissatisfaction because of adoption of certified EHR. To our knowledge, this is the first study that provides empirical evidence at hospital level using national data to support that policy makers should advocate incentive programs in psychiatric hospitals. Federal and state governments should expand the incentive program to psychiatric providers for the certified EHR adoption and sponsor to update certified EHR to meet the requirements of psychiatric privacy laws. [50, 51]

There are limitations to our study that should be noticed. First, we excluded hospitals with missing values in key variables when merging the data from different data sources. This may create selection bias. There may also be selection bias in patients that chose to respond to the HCAHPS survey. [52] Second, as an observational study, it cannot identify the causal mechanisms underlying the relationships between patient perceptions and certified EHR adoption, although we adjusted for several potential confounders (e.g., teaching status, location, ownership). In addition, our findings may not be generalizable to non-psychiatric hospitals or outpatient settings. Finally, analysis of this study at hospital level did not consider patient demographic characteristics which were not available from the original data but may potentially inform patient experience and bring about bias.

Further study should apply longitudinal national data to testify about the causal association between certified EHR and patient experience, with considering more potential confounders like patient characteristics. Also, intrinsic mechanism should be examined to explain how certified EHR adoption is able to improve overall patient evaluation in psychiatric hospitals.

## Summary

In conclusion, we found marked variation in patient experience between hospitals with and without certified EHR across U.S. psychiatric hospitals. Psychiatric hospitals with certified EHR are more likely to get higher level of patient experience. While results of this study do not imply causality between patient experience and certified EHR adoption, they suggest the positive association between certified EHR adoption and patient experience.
